# Different anti-adipogenic effects of bio-compounds on primary visceral pre-adipocytes and adipocytes

**DOI:** 10.17179/excli2016-267

**Published:** 2016-06-20

**Authors:** Monica Colitti, Bruno Stefanon

**Affiliations:** 1 Department of Scienze Agroalimentari, Ambientali e Animali, University of Udine, via delle Scienze, 206, 33100 Udine, Italy

**Keywords:** natural compounds, apoptosis, differentiation, lipolysis, visceral adipocytes

## Abstract

Several natural compounds exhibit strong capacity for decreasing triglyceride accumulation, enhancing lipolysis and inducing apoptosis. The present study reports the anti-adipogenic effects of *Silybum marianum *(SL), *Citrus aurantium* (CA), *Taraxacum officinale* (TO), resveratrol (RE), *Curcuma longa* (CU), caffeine (CF), oleuropein (OL) and docosahexaenoic acid (DHA) in reducing differentiation and increasing lipolysis and apoptosis. Analyses were performed on human primary visceral pre-adipocytes after 10 (P10) and 20 (P20) days of treatment during differentiation and on mature adipocytes after 7 days of treatment (A7). The percentage of apoptosis induced by TO extract in P10 and P20 cells was significantly higher than that induced by all other compounds and in CTRL cells. Triglyceride accumulation was significantly lower in cells treated with DHA, CF, RE in comparison to cells treated with OL and in CTRL cells. Treatments with CF, DHA and OL significantly incremented lipolysis in P20 cells in comparison to other compounds and in CTRL cells. On the contrary, the treatment of A7 cells with OL, CA and TO compounds significantly increased cell lipolysis. The addition of CF in differentiating P20 pre-adipocytes significantly increased the expression of genes involved in inhibition of adipogenesis, such as GATA2, GATA3, WNT1, WNT3A, SFRP5, and DLK1. Genes involved in promoting adipogenesis such as CCND1, CEBPB and SREBF1 were significantly down-regulated by the treatment. The screening of bioactive compounds for anti-adipogenic effects showed that in differentiating cells TO extract was the most effective in inducing apoptosis and CF and DHA extracts were more efficient in inhibition of differentiation and in induction of cell lipolysis.

## Abbreviations

ACACB, acetyl-CoA carboxylase beta; ADIG, adipogenin; ADIPOQ, adiponectin; ADRB2, adrenoceptor beta 2; AGT, angiotensinogen; ANGPT2, angiopoietin 2; AXIN1, axin 1; BMP2, bone morphogenetic protein 2; BMP4, bone morphogenetic protein 4; BMP7, bone morphogenetic protein 7; CCND1, cyclin D1; CDK4, cyclin-dependent kinase 4; CDKN1A, cyclin-dependent kinase inhibitor 1A (p21, Cip1); CDKN1B, cyclin-dependent kinase inhibitor 1B (p27, Kip1); CEBPA, CCAAT/enhancer binding protein (C/EBP) alpha; CEBPB, CCAAT/enhancer binding protein (C/EBP) beta; CEBPD, CCAAT/enhancer binding protein (C/EBP) delta; CFD, complement factor D (adipsin); CREB1, cAMP responsive element binding protein 1; DDIT3, DNA-damage-inducible transcript 3; DIO2, deiodinase, iodothyronine, type II; DKK1, dickkopf WNT signaling pathway inhibitor 1; DLK1, delta-like 1 homolog (Drosophila); E2F1, E2F transcription factor 1; EGR2, early growth response 2; FABP4, fatty acid binding protein 4, adipocyte; FASN, fatty acid synthase; FGF1, fibroblast growth factor 1 (acidic); FGF10, fibroblast growth factor 10; FGF2, fibroblast growth factor 2 (basic); FOXC2, forkhead box C2 (MFH-1, mesenchyme forkhead 1); FOXO1, forkhead box O1; GATA2, GATA binding protein 2; GATA3, GATA binding protein 3; HES1, hes family bHLH transcription factor 1; INSR, insulin receptor; IRS1, insulin receptor substrate 1; IRS2, insulin receptor substrate 2; JUN, jun proto-oncogene; KLF15, Kruppel-like factor 15; KLF2, Kruppel-like factor 2; KLF3, Kruppel-like factor 3; KLF4, Kruppel-like factor 4; LEP, leptin; LIPE, lipase, hormone-sensitive; LMNA, lamin A/C; LPL, lipoprotein lipase; LRP5, low density lipoprotein receptor-related protein 5; MAPK14, mitogen-activated protein kinase 14; NCOA2, nuclear receptor coactivator 2; NCOR2, nuclear receptor corepressor 2; NR0B2, nuclear receptor subfamily 0, group B, member 2; NR1H3, nuclear receptor subfamily 1, group H, member 3; NRF1, nuclear respiratory factor 1; PPARA, peroxisome proliferator-activated receptor alpha; PPARD, peroxisome proliferator-activated receptor delta; PPARG, peroxisome proliferator-activated receptor gamma; PPARGC1A, peroxisome proliferator-activated receptor gamma, coactivator 1 alpha; PPARGC1B, peroxisome proliferator-activated receptor gamma, coactivator 1 beta; PRDM16, PR domain containing 16; RB1, retinoblastoma 1; RETN, resistin; RUNX1T1, runt-related transcription factor 1; RXRA, retinoid X receptor, alpha; SFRP1, secreted frizzled-related protein 1; SFRP5, secreted frizzled-related protein 5; SHH, sonic hedgehog; SIRT1, sirtuin 1; SIRT2, sirtuin 2; SIRT3, sirtuin 3; SLC2A4, solute carrier family 2 (facilitated glucose transporter), member 4; SRC, v-src avian sarcoma (Schmidt-Ruppin A-2) viral oncogene homolog; SREBF1, sterol regulatory element binding transcription factor 1; TAZ, tafazzin; TCF7L2, transcription factor 7-like 2 (T-cell specific, HMG-box); TSC22D3, TSC22 domain family, member 3; TWIST1, twist family bHLH transcription factor 1; UCP1, uncoupling protein 1 (mitochondrial proton carrier); VDR, vitamin D (1,25- dihydroxyvitamin D3) receptor; WNT1, wingless-type MMTV integration site family, member 1; WNT10B, wingless-type MMTV integration site family, member 10B; WNT3A, wingless-type MMTV integration site family, member 3A; WNT5A, wingless-type MMTV integration site family, member 5A; WNT5B, wingless-type MMTV integration site family, member 5B.

## Introduction

The remodeling of adipose tissue is a dynamic process that is related to adipogenesis and apoptosis. The associated changes in adipocytes number is regulated by the expression of a battery of genes which controls cell fate. In accordance with Rayalam et al. (2008[48]), bioactive components of plant extracts can have direct effects on adipose tissue. According to the Food and Drug Administration (FDA) a dietary supplement should contain one or many ingredients among vitamins, minerals, herbs, amino acids, concentrates, metabolites or extracts (Ross, 2000[49]). Instead, a nutraceutical differs since it must not only supplement the regular diet, but also aid in the prevention of treatment of disease and disorder (Kalra, 2003[31]). In this regard, natural anti-obesity molecules can reduce body weight by decreasing lipid absorption, energy intake, pre-adipocyte differentiation and proliferation and lipogenesis or increasing the energy expenditure and lipolysis (Yun, 2010[64]). As extensively reviewed by Colitti and Grasso (2014[14]), several nutraceuticals have been considered as anti-obesity molecules for their capacity to decrease triglyceride accumulation, enhance lipolysis, induce apoptosis or modify gene expression. In recent years, apoptosis was considered one of the main targets to reduce the number of pre-adipocytes or adipocytes thus preventing hyperplasia and hypertrophia (Rayalam et al., 2008[48]; Herold et al., 2013[27]). 

Silymarin, a compound of the crude extract of plant milk thistle plant* (Silybum marianum *SL*), *is known for its hepatoprotective effect and chemopreventive activites (Raina and Agarwal, 2007[47]). Silymarin was also found to attenuate adipogenesis and to decrease glucose uptake, directly inhibiting GLUT4-mediated glucose transport (Ka et al., 2009[30]; Zhan et al., 2011[65]).

The bitter orange extract of *Citrus aurantium* (CA) fruits contains protoalkaloids, the most representative being p-synephrine (known also as oxedrine), which is used for the treatment of obesity. The extract also contains other protoalkaloids as octopamine, hordenine and tyramine (Stohs et al., 2011[55]). Also flavonoids, such as limonene, hesperidin, neohesperidin and naringin, together with furanocoumarins are typically present in CA fruit extract (Wichtl, 2004[62]). Octopamine can induce the lipolysis of adipocytes in rat, hamster and dog through the agonistic activity on the β-adrenergic receptor, even though this effect was not confirmed not on human adipocytes (Carpéné et al., 1999[8]). Instead, studies on human subcutaneous fat cells have been demonstrated that synephrine has a lipolytic action (Mercader et al., 2011[39]). A recent study conducted on 3T3-L1 cells treated with of CA extracts demonstrated its anti-adipogenic activity through inhibition of Akt and downregulation of C/EBPβ (Kim et al., 2012[33]).

*Taraxacum officinale* (TO) plant of the genus *Taraxacum* has been used for a long time in many traditional herbal medical systems to treat dyspepsia, heartburn, spleen and liver complaints, hepatitis and anorexia. It has been reported that TO has also diuretic, anti-inflammatory, anti-oxidative, anti-carcinogenic, analgesic, anti-hyperglycemic and anti-coagulatory properties (Schütz et al., 2006[51]). A decrease in body weight was observed in mice fed with high fat diet supplemented with TO leaf extract. Moreover, TO leaf extract supplement suppressed serum levels of triglyceride, total cholesterol and insulin induced by the high fat diet (Davaatseren et al., 2013[15]).

Caffeine (CF), a xanthine alkaloid, is contained in many plants such as *Coffea canephora*, various tea brush and yerba maté (*Ilex paraguariensis*) (Yun, 2010[64]). This natural compound reduces accumulation of lipids in adipocytes and enhances lipolysis by inhibiting phosphodiesterase activity (Herman and Herman, 2013[26]). A noradrenaline-like mechanism able to induce lipolysis was hypothesized for CF at low concentration. The subsequent stimulation of β-adrenergic receptors led to an increase of cytosolic cAMP and so activates protein kinase A (Han et al., 1999[25]). Finally, on 3T3-L1 cells, CF allowed differentiation, but reduced lipid accumulation on mature adipocytes. Moreover, CF prevented insulin-dependent glucose uptake in mature adipocytes (Nakabayashi et al., 2008[41]).

Curcumin (CU) is extracted from *Curcuma longa,* a widely used spice in Asia. This polyphenol is used to treat cancer, aging, endocrine, immunological, gastrointestinal and cardiac diseases. The CU extract is active also on oxidative stress, inflammation, and cell death pathways (Witkin and Li, 2013[63]). It is demonstrated on 3T3-L1 cells that CU decreases differentiation of pre-adipocytes and reduces accumulation of lipids in already mature adipocytes both *in vivo* and *in vitro*. 

Resveratrol (RE) is a stilbenoid originally found in the berries of the wine grape. In the last years it is the most investigated among all other phytochemicals. Recent studies demonstrated that resveratrol is able to prevent adipogenesis in 3T3-L1 cells (Hwang et al., 2010[28]; Chen et al., 2011[11]; Vigilanza et al., 2011[60]) through the down-regulation of proteins related with fatty acid metabolism, such as fatty acid synthase (FAS) and acetyl-CoA carboxylase (ACAC) (Hwang et al., 2010[28]). On human mature adipocytes at a concentration of 100µM, RE was able to induce lipolysis and impaired lipogenesis (Gomez-Zorita et al., 2013[20]). A possible effect of resveratrol on apoptosis was also proposed in a recent research. Studies demonstrated that resveratrol inhibits the phosphorylation of protein kinase B (Akt), which activates Bax and consequently the caspase-dependent intrinsic pathway of apoptosis (Rayalam et al., 2008[48]).

Oleuropein (OL) is the most abundant phenolic compound that is found in olive pulp, seed, leaves, and peel of unripe olives, where can constitute up to 14 % of the dry weight (Barbaro et al., 2014[4]). Oleuropein supplementation reduces body, liver and heart weights in high fat diet-treated mice (Poudyal, et al., 2010[46]). On 3T3-L1 cells was shown that OL treatment reduces pre-adipocyte differentiation and lipid accumulation by decreasing the expression of PPARγ and C/EBPα and their downstream target genes (Drira et al., 2011[17]).

Docosahexaenoic acid (DHA), a (n-3) PUFA present in fish oil, has been demonstrated to inhibit adipocyte differentiation. The suppression of fatty acid synthesis and the regulation of adipocytes differentiation can be regulated by polyunsaturated fatty acids (PUFAs) (Madsen et al., 2005[38]; Okuno et al., 1997[43]). This is probably due to the more difficult acetylation process of PUFA compared to the acetylation of monounsaturated fatty acids. Moreover, PUFA act as signal transducing molecules in adipocytes (Okuno et al., 1997[43]; Awad et al., 2000[2]; Evans et al., 2000[18]). The treatment with DHA of 3T3-L1 cells for 48 h negatively affected droplet size and percentage of lipids in a concentration-dependent manner (Kim et al., 2006[34]). Addition of DHA (50-200 µM) to fully differentiated 3T3-L1 adipocytes increased basal lipolysis by inducing glycerol release (Kim et al., 2006[34]). Moreover, it has been observed that DHA (100 µM) treatment increased lipolysis in 3T3-L1 cells by an up-regulation of adipose triglyceride lipase (ATGL) and a down-regulation of perilipin gene expression (Barber et al., 2013[5]). Other studies on 3T3-L1 cells demonstrated that DHA inhibits cell proliferation as well (Awad et al., 2000[2]).

The present study describes the anti-adipogenic effects of *Silybum marianum* (SL), *Citrus aurantium* (CA), *Taraxacum officinale* (TO), resveratrol (RE),* Curcuma longa* (CU), caffeine (CF), oleuropein (OL) and docosahexaenoic acid (DHA) in inhibition of the pre-adipocyte differentiation and in induction of the cell lipolysis and apoptosis in human primary visceral adipocytes. Moreover, the effect of CF on the differential expression of genes involved in adipogenesis are reported. 

## Material and Methods

### Cells

Human omental pre-adipocyte cells and medium were obtained from ZenBio (USA). According to the data kindly provided by ZenBio, pre-adipocyte cells (OP-F-3) were sampled from three Caucasian donor females, with the mean age of 48.7 ± 9. 1 years and the mean BMI of 42. 7 ± 6. 9 kg/cm^2^. Donors were non-diabetic and non-smoking. Visceral pre-adipocytes were plated and proliferated in Omental Preadipocyte Medium (OM-PM) till the passage 3. All the experiments were run in the humidified 37 °C incubator with 5 % CO_2_. OM-PM was changed each second day till the cells reached full confluence. At this time OM-PM was completely removed, cells were washed with phosphate buffered saline (PBS 1X) and fed with Omental Differentiation Medium (OM-DM) and further with Omental Adipocyte Medium (OM-AM) added with compounds at a concentration of 5, 10, 30 and 70 µg/mL with final concentration of 0.0014 % dimethyl sulfoxide (DMSO). The control cells (CTRL) were incubated in OM-DM and OM-AM without compounds in the present of 0.0014 % DMSO as well.

Treatments were performed from the first day of differentiation till 10 days in OM-DM (P10), till 20 days in OM-DM (P20) and during 7 days post differentiation in OM-AM (A7). Cells were analyzed at the end of each differentiation period.

Apoptosis, adipogenesis and lipolysis assays were performed in a 96 well plate with the cell density of 10^4^ cells/well, while PCR assay was performed in a 6 well plate with the cell density of 10^5^ cells/well. All analyses were performed using cells of three different donors and each donor was assayed in triplicate.

### Cell treatments

Cells were treated with the following plant extracts: *Citrus aurantium* (CA), *Silybum marianum* (SL) and *Taraxacum officinale* (TO), resveratrol (RE) *Curcuma longa* (CU), provided by ACEF spa (Piacenza, Italy). Cells were also tested with the following bioactive compounds: caffeine (CF), oleuropein (OL) and docosahexaenoicacid (DHA) purchased by Sigma (Table 1(Tab. 1)). Extracts and compounds were collectively named compounds if not differentially specified.

All compounds were suspended in 10 % dimethyl sulfoxide (DMSO), with the exception of DHA that was suspended in 100 % ethanol. 

### Cell viability assay

The cytotoxicity of compounds at different doses (5, 10, 30 and 70 μg/mL) was measured using the MTT colorimetric assay (3-(4,5-dimethylthiazol-2-yl)-2,5-diphenyltetrazolium bromide) (Pomari et al., 2015[45]). At the end of each differentiation period (P10, P20, A7) cells were washed with PBS, added with MTT solution (5 mg/mL in fresh medium) and incubated for 3 hours at 37 °C. Then, the mixture was carefully removed from each well followed by addition of 100 µL of DMSO. Absorbance was read with a microplate reader at 570 nm. The percentage of viable cells was calculated by normalizing the absorbance value of the treated cells by the absorbance value of the CTRL cells. 

### Apoptosis assay

Detection of apoptotic cells was performed using ApoStrand^TM^ ELISA apoptosis detection kit (Enzo Life Sciences Inc., NY, USA) according to manufacturer's instructions (Pomari et al., 2015[45]). The apoptotic positive control (single stranded DNA in PBS) was also included in the analysis. Data are expressed as the percentage of cells, comparing the optical density of the treated/CTRL cells with the optical density of the positive CTRL included in the kit.

### Adipogenesis assay

Intracellular lipid accumulation in P10 and P20-treated and in CTRL cells was analyzed by microscopy (PrimoVert, Zeiss, Jena Germany) after staining cells with Oil red O (ORO, Sigma, Milan, Italy), and quantified using a spectrophotometer at 520 nm. Data are expressed as percentage of lipid accumulation relative to the CTRL samples.

### Lipolysis assay

Detection of the lipolytic activity in P20 and A7 treated cells was performed using AdipoLyze^TM^ Lipolysis Detection Kit (Lonza Walkersville Inc., MD, USA), based on the quantification of the glycerol release by cells undergoing lipolysis (Pomari et al., 2015[45]). Accumulated glycerol in each sample was determined by comparison with a glycerol standard curve.

### RNA extraction and adipogenesis PCR array on CF-treated P20 cells

Prior to the total RNA extraction CF-treated P20 and CTRL cells were rinsed with ice-cold PBS followed by the RNA extraction with miRNeasy kit and QIAzol Lysis Reagent (Qiagen, Milan, Italy) according to the manufacturer's recommendations. Synthesis of the first strand cDNA was performed using RT^2^ First Strand kit (Qiagen, Milan, Italy) (Stefanon et al., 2015[54]). 

The gene expression profile of adipogenesis was determined using ready to use human Adipogenesis RT^2^ Profiler PCR Array (PAHS-049Z; Qiagen, Milan, Italy) containing primers for 84 tested, 5 housekeeping genes and controls for RT and PCR reactions. The expression of target genes was normalized and ΔCts were calculated by the difference between Ct of target genes and the geometric mean of the four housekeeping genes. Differences between CF-treated P20 cells and P20 CTRL cells were calculated using the 2^-ΔΔCt^ method (Livak and Schmittgen, 2001[36]; Bustin et al., 2009[7]), where 2^-ΔΔCt^ represents the difference of a given target gene in CF-treated cells *vs* CTRL. The *n*-fold expression of a given target gene was calculated as log_2_(2^-ΔΔCt^).

### Statistical analysis

In the cell viability assay, differences between treated cells were determined by one-way ANOVA with the concentration (4 levels) as fixed factor (SPSS®, 1997[53]). Significant effects of the treatments on cell apoptosis, triglyceride accumulation and lipolysis were assessed with two-way ANOVA considering the fixed effect of treatment (treated and CTRL cells) and the fixed effect of period of differentiation (for apoptosis: P10, P20 and A7 cells; for lipolysis assay: P20 and A7 cells; for lipid accumulation assay: P10 and P20 cells). The effect of interaction (treatment x period of differentiation) was tested in two-way ANOVA as well.

PCR array data, expressed as log_2_(*n*-fold), were analysed using one sample *T*-test (SPSS®, 1997[53]) and *P* values adjusted for multiple testing with false discovery rate (FDR). Results are displayed in a form of a Volcano plot, which graphically reports the relationships of the fold changes with statistical values calculated as the negative Log10 (*P*-value). In the plot, the horizontal red line illustrates a significant cutoff of *P* = 0.05.

## Results

### Cell viability

A viability assay was used to find out the highest dose of extracts that allowed the cell viability to remain over 60 %. Treatment of P10, P20 and A7 cells with 5, 10, 30 or 70 µg/mL compounds, or 25, 50, 100, 200 μM DHA showed that cell viability of P20 cells significantly (*P* < 0.001) decreased in a dose-dependent manner (Table 2(Tab. 2)). In P10 and P20 treated cells the viability remained over 70 % and 60 % respectively until a compound concentration of 30 µg/mL. At a dose of 70 µg/mL viability was always significantly different from other doses (*P* < 0.001). The viability of A7 treated cells remained over 90 % with the dose up to 70 µg/mL for all compounds, except RE. Different doses of DHA showed the same significant (*P* < 0.001) dose-dependent decrease in P20 treated cells, but the cells' viability at P10 and P20 remained over 60 %. According to these evidences, the concentration of 30 µg/mL of compounds and 50 μM DHA was chosen for the experiments.

### Effect on apoptosis

**Figure 1:**
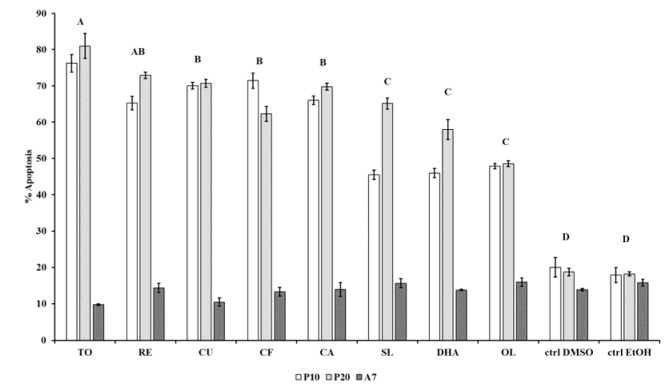
Modulation of apoptosis by all compounds in human omental pre-adipocytes and mature adipocytes. P10, differentiating pre-adipocytes treated for 10 d; P20, differentiating pre-adipocytes treated for 20 d; A7, mature adipocytes treated for 7 d. Data are presented as mean percentage ± standard error (SE) *vs* positive CTRL. Capital letters above bars indicate the significant difference for *P* < 0.001 between the mean percentage of apoptosis of the treatments. Capital letter A indicates that TO treatment significantly differs from CU, CF, CA, SL, DHA, OL treatments and CTRLs; capital letter AB indicates that RE treatment significantly differs from SL, DHA, OL treatments and CTRLs; capital letter B indicates that CU, CF and CA treatments significantly differ from TO, SL, DHA, OL and CTRLs; capital letter C indicates that SL, DHA and OL treatments significantly differ from TO, RE, CU, CF, CA and CTRLs; capital letter D indicates that CTRLs significantly differ from TO, RE, CU, CF, CA, SL, DHA, OL. The mean percentage of apoptosis between P10, P20 and A7 is significantly different (*P* < 0.001) as well. Compound: SL, *Silybum marianum*, CA, *Citrus aurantium*, RE, Resveratrol, TO, *Taraxacum officinale*, CU, *Curcuma longa*, CF, Caffeine, OL, Oleuropein, DHA, Docosahexaenoicacid

The apoptotic effect of the compounds was examined in P10, P20 and A7 cells treated with 30 μg/mL extracts or 50 µM DHA and in corresponding CTRL cells. A significant (P < 0.001) difference of the percentage of apoptosis in P10, P20 and A7 cells under treatments was observed (Figure 1(Fig. 1)).

The interaction of cell apoptosis between different stages and treatments was also significant (P < 0.001). Interestingly, the percentage of apoptosis induced by TO extract (76.19 ± 2.4 % at P10 and 81.0 ± 3.4 % at P20) was significantly (P < 0.001) higher than that induced by all other extracts and in CTRL cells. Among the compounds, SL, DHA and OL were significantly (P < 0.001) less active in inducing apoptosis.

### Effect on adipogenesis

The percentage of apoptosis was affected by treatments and was different between mature adipocytes (A7) and differentiating cells. Therefore, the ability of compounds to prevent triglyceride accumulation in P10 and P20 pre-adipocytes in comparison to CTRL cells was evaluated in order to verify the anti-adipogenic effect of studied compounds only during differentiation.

**Figure 2:**
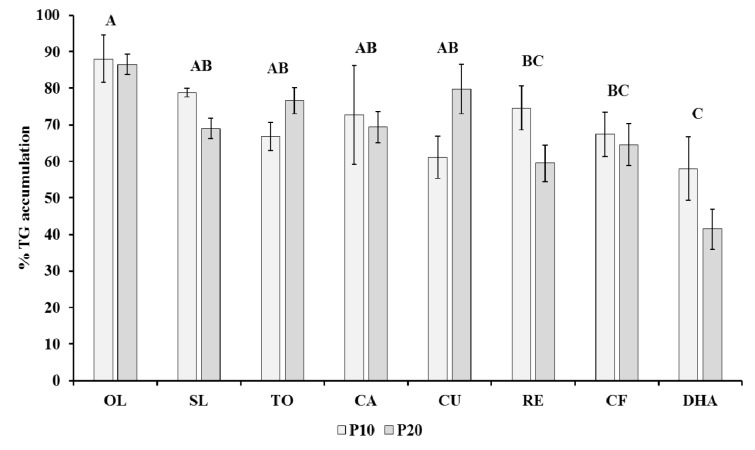
Effects of compounds on triglyceride increment during pre-adipocytes differentiation. Triglyceride accumulation of differentiating pre-adipocytes incubated for 10 d (P10) and 20 d (P20) with compounds relative to untreated control cells (CTRL) settled at 100 %. Results are depicted as mean percentage ± standard error (SE). Capital letters above bars indicate the significant difference for *P* < 0.001 between the mean percentage of the treatments. Capital letter A indicates that OL treatment significantly differs from RE, CF and DHA treatments; capital letter AB indicates that OL, SL, TO, CA, CU, RE and CF treatments significantly differ from DHA treatment; capital letter BC indicates that RE and CF treatments significantly differ from OL treatment; capital letter C indicates that DHA treatment significantly differs from OL, SL, TO, CA, CU, RE, CF treatments. The percentage of TG accumulation in P10 cells is not significantly different in comparison to P20 cells. Compound: SL, *Silybum marianum*, CA, *Citrus aurantium*, RE, Resveratrol, TO, *Taraxacum officinale*, CU, *Curcuma longa*, CF, Caffeine, OL, Oleuropein, DHA, Docosahexaenoicacid

Analysis was conducted in P10 and P20 cells treated with 30 µg/mL extracts and 50 µM DHA and in the corresponding CTRL cells. The amount of triglyceride accumulation was apprised as a percentage respect to CTRL, considered as 100 % (Figure 2(Fig. 2)). Received data indicate that adipogenesis was inhibited by all tested compounds in P10 and P20 cells, showing a significant (*P* < 0.001) decrease of triglyceride accumulation in comparison to CTRL cells. Triglyceride accumulation was significantly (*P* < 0.001) lower in cells treated with DHA, CF, RE (57.99 ± 6.0, 67.43 ± 7.9, 74.58 ± 8.7 in P10 cells and 41.44 ± 3.0, 64.6 ± 6.2, 59.48 ± 5.4 in P20 cells) in comparison to cells treated with OL (88.04 ± 3.9 in 10 cells and 86.54 ± 3.5 in P20 cells). The effect of days of incubation was not significantly different as well as the interaction between days of incubation and compounds.

### Effect on lipolysis

**Figure 3:**
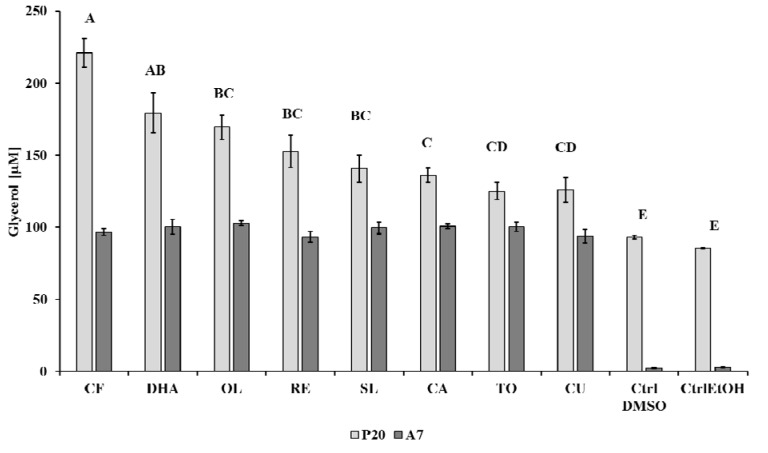
Determination of glycerol release (μM) in differentiating (P20) and mature (A7) adipocytes after incubation with all studied compounds. Results are depicted as mean (μM) ± standard error (SE). Capital letters above bars indicate the significant difference for *P* < 0.001 of the mean of glycerol release (μM) between the treatments. Capital letter A indicates that CF treatment significantly differs from OL, RE, SL, CA, TO, CU treatments and CTRLs; capital letter AB indicates that DHA treatment significantly differs from CA, TO, CU treatments and CTRLs; capital letter BC indicates that OL, RE and SL treatments significantly differ from CF and CTRLs; capital letter C indicates that CA treatment significantly differs from CF, DHA and CTRLs; capital letter CD indicates that TO and CU treatments significantly differ from CF, DHA and CTRLs; capital letter E indicates that CTRLs significantly differ from CF, DHA, OL, RE, SL, CA, TO, CU treatments. The glycerol release in P20 cells is significantly different (*P* < 0.001) from A7 cells. Compound: SL, *Silybum marianum*, CA, *Citrus aurantium*, RE, Resveratrol, TO, *Taraxacum officinale*, CU, *Curcuma longa*, CF, Caffeine, OL, Oleuropein, DHA, Docosahexaenoicacid

To assess if lipolysis is affected by compounds, we measured glycerol release in P20 pre-adipocytes, when cells were still in active development, but TG accumulation is reduced in comparison to P10 cells, and on mature cells (A7), which contain large lipid droplets in the cytoplasm and present rounded shape. Treatment of P20 cells with CF, DHA and OL extracts significantly (P < 0.001) incremented the content of free glycerol in the culture medium respectively to 221.0 μM (± 9.97), 179.0 μM (± 13.8) and 169.4 μM (± 8.4) as compared to other compounds and CTRL cells (Figure 3(Fig. 3)). The mean free glycerol of TO (125.0 μM ± 5.8) and CU (126.0 μM ± 8.6) treated cells significantly (P < 0.001) differed from cells treated with other compounds showing a decrease of release in the culture media. On the contrary, the treatment of A7 cells significantly (P < 0.001) increased the release of free glycerol, in particular with OL (102.5 ± 1.6 μM), CA (100.4 ± 1.7 μM) and TO (100.3 ± 3.4 μM) compounds (Figure 3(Fig. 3)). The interaction between different cell stages of differentiation and treatments was significant (P < 0.001).

### Effects of CF on the gene expression of adipogenesis-associated genes

**Figure 4:**
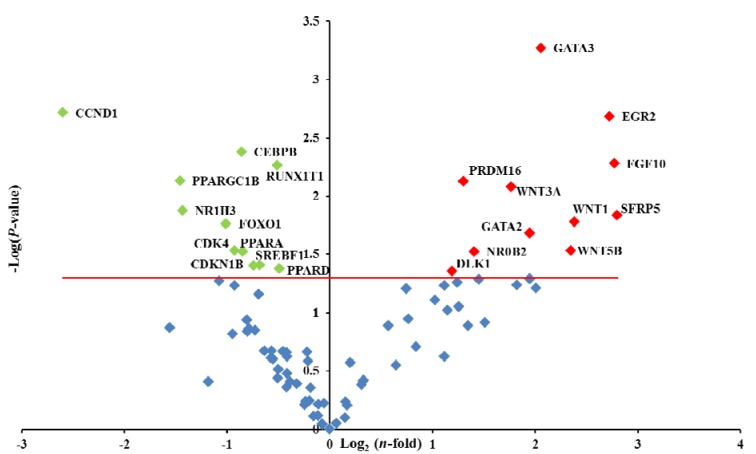
Volcano plot of adipogenesis human RT^2^ Profiler PCR array. PCR array analysis of gene expression in 30 μg/mL CF-treated P20 cells in comparison to P20 CTRL cells. The relative expression levels for each gene depicted as log_2_(*n*-fold) are plotted against -Log_10_(*P*-value). Red indicator = significantly up-regulated gene; Green indicator = significantly down-regulated gene. Red line = indicates -Log_10_(*P* -value), *p* < 0.05. Treatment: CF, caffeine

The CF was the most efficient compound in decreasing TG accumulation and the most efficient in stimulating lipolysis. Taking in account that the influences of CF on adipocyte differentiation has not been yet completely elucidated, the expression pattern of genes involved in the adipogenesis pathways was measured using the human RT^2^ Profiler PCR Array on CF-treated P20 cells. Volcano plot reported the log_2_(*n-*fold) values of significantly (*P* < 0.05) up- and down-regulated genes in comparison to CTRL cells (Figure 4(Fig. 4)). 

The results revealed that 30 μg/mL of CF compound significantly (*P* < 0.05) up-regulated the expression 11 genes (13 %) and down-regulated 11 genes (13 %) (Figure 4(Fig. 4)).

Among the genes that negatively affected adipogenesis DLK1, GATA2, GATA3, PRDM16, SFRP5, WNT1 and WNT3A were significantly (*p *< 0.05) up-regulated in P20 cells treated with CF extract. Instead, CCND1, CDK4, CDKN1B, NR1H3, PPARA, PPARD, PPARGC1B, and SREBF1 were significantly (*P *< 0.05) down-regulated in the P20 cells treated with CF extract.

## Discussion

In this study, nine different bioactive compounds were assayed for their ability to influence the apoptosis, adipogenesis and lipolysis in primary visceral human pre-adipocytes and adipocytes. 

DHA, RE and CF were the significantly strongest inhibitors of adipogenesis as evidenced by the percentage of triglyceride accumulation (Figure 2(Fig. 2)). Present data are in agreement with Kim et al. (2006[34]) who demonstrated that DHA reduced lipid accumulation in 3T3-L1 cells by inducing apoptosis and promoting lipolysis. However, in the present study, the lipolytic effect was more evident during differentiation (P20) than in mature adipocytes (A7), as also reported on 3T3-L1 (Kim et al., 2008[35]). Interestingly, as evidenced by previous studies on P20 cells treated with *Rosmarinus* *officinalis* (Stefanon et al., 2015[54]) and *Rhodiola rosea* (Pomari et al., 2015[45]), the reduction of triglyceride incorporation during differentiation was coincident with an enhancement of lipolytic activity mediated by DHA and CF.

RE at the concentration of 30 µg/mL, is known to suppress adipogenesis in 3T3-L1 cells in comparison to CTRL cells (Santos et al., 2014[50]). It is also demonstrated that RE extract induces apoptosis in 3T3-L1 pre-adipocytes, adipocytes and in rat primary adipocytes (Rayalam et al., 2008[48]). In the present study RE was found to exert its apoptotic function only in P20, but not in A7 cells (Figure 1(Fig. 1)). Of note, neither one compound was able to promote apoptosis in mature cells. 

Silibinin, the major active constituent of SL, has been recognized to inhibit adipocyte differentiation by inducing cell cycle arrest in the G0/G1 phase through the control of cell cycle regulators (Suh et al., 2015[56]). SL-treated P10 and P20 cells showed respectively a 78.8 ± 13.5 % and 69.0 ± 4.2 % of TG accumulation in comparison to P20 CTRL cells and SL-treatment was significantly different from the DHA treatment (Figure 2(Fig. 2)). Other evidences suggested that the decrease of adipogenesis in human differentiated adipocytes treated with silibinin was due to the promotion of a brown remodeling that, in comparison to control cells, was around 74 % (Barbagallo et al., 2016[3]), a value quite similar to present results. OL was the less active compound in reducing adipogenesis, although on 3T3-L1 cells it has been shown that OL exerts its anti-adipogenic effect during the first 2 days of differentiation by arresting or delaying the cell cycle (Drira e al., 2011[17]). On the other hand, OL was the compound that in P20 cells exhibited the strongest effect on apoptosis together with SL and DHA treatments (Figure 1(Fig. 1)). These results are consistent with the data received on MCF-7 cancer cells (Han et al., 2009[24]).

There is little information available concerning the anti-obesity effect of dandelion and most of the literature consists of *in vivo* studies (Gamboa-Gómez et al., 2015[19]). As reviewed by Schütz et al. (2006[51]), TO contains a wide array of phytochemicals distributed along the plant. In the leaves of dandelion are mostly present terpenes and phenolic compounds including coumaric acid, chicoric acid, monocaffeoyltartaric acid, cinnamic acid, caffeic acid, chlorogenic acid, and luteolin (González-Castejón et al., 2012[21]). Among the effects studied on TO-treated P10, P20 and A7 cells, apoptosis was the most effective, respect to its effect on the TG accumulation and lipolysis. This is consistent with the strong induction of apoptosis observed on pancreatic cancer cells treated with dandelion root extract (Ovadje et al., 2012[44]). Since in the flowers and leaves the most abundant phenolic compounds are caffeic acid esters, such as chlorogenic acid, dicaffeoyltartaric (chicoric) acid and monocaffeoyltartaric acid, the observed effect of TO on differentiation can be comparable to that of CF.

It is known that CF stimulates hydrolysis of TG through the activation of HSL and β-adrenergic receptors (de Matteis et al., 2002[16]). On 3T3-L1 cells was shown that caffeine (> 1 mM) suppresses adipocytes differentiation by inhibition of C/EBPα and PPARγ expression, known as the main adipogenic transcription factors, and by promotion of the expression of negative regulators such as pre-adipocyte factor 1 (Pref-1, also known as DLK1) and KLF2 (Kim et al., 2016[32]). 

Considering the results of cell differentiation and lipolysis obtained from the CF treatment, the comparative gene expression analysis on P20 CF-treated and CTRL cells was performed (Figure 4(Fig. 4)). GATA2, GATA3, DLK1, WNT1 and WNT3A are among the genes significantly up-regulated by CF- treatment, which are known as strong inhibitors of adipogenesis (Lowe et al., 2011[37]; Moreno-Navarrete and Fernández-Real, 2012[40]). GATA2 and GATA3, two zinc-finger DNA-binding proteins transcription factors are typically expressed in pre-adipocytes and down-regulated during development. It was shown that GATA2 and GATA3 are able to bind PPARγ promoter thus preventing its transcription and blocking cells at the pre-adipocyte stage (Tong et al., 2000[59]). DLK1, known as pre-adipocyte factor 1 (Pref-1), is also highly expressed in pre-adipocytes and normally declines during differentiation. The final target of the pathway downstream of DLK1 is Sox9, a transcription factor expressed in pre-adipocytes to suppress expression of C/EBPβ and C/EBPδ. Pref-1 prevents down-regulation of Sox9, which is required before adipocyte differentiation can proceed, resulting in inhibition of adipogenesis (Sul, 2009[57]). In fact, in CF-treated P20 cells, expression of CEBPB gene was found to be significantly down-regulated as well as expression of SREBF1 gene (Figure 4(Fig. 4)). The down-regulation of this transcription factor in turn reduced the expression of most of the genes involved in lipid accumulation and adipocyte differentiation. Many compounds, such as Chinese herbal medicine Yi-Gan-San and Vitisin A, have been described as inhibitors of adipogenesis *via* SREBF1 regulation (Izumi et al., 2009[29]; Kim et al., 2008[35]). Recently on 3T3-L1 pre-adipocytes was demonstrated that epiberberine, a natural alkaloid from the rhizome of *C. chinensis*, down-regulated protein expression of SREBF1 through the inhibition of Raf/MEK1/ERK1/2 and AMPKa1/Akt phosphorylation (Choi et al., 2015[12]). SREBF1 is also inhibited by betulin, promoting the interaction between SCAP (SREBF chaperone) and INSIG (insulin induced gene) which leads to the endoplasmic reticulum-retention of SREBP (Tang et al., 2011[58]).

CDK4 is an important regulator of adipocyte differentiation, promoting adipogenesis through PPARG activation (Abella et al., 2005[1]). In CF-treated P20 cells CDK4 was down-regulated and this agrees with the fact that CDK4 inactivation blocks adipocyte differentiation (Chavey et al., 2013[10]). 

WNTs are a large family of extracellular effectors which are known to play an important role in the suppression of adipogenesis through the canonical WNT signaling pathway (Gustafson and Smith, 2010[23]), although other mechanisms have been proposed (Sethi and Vidal-Piug, 2010[52]). Nevertheless, all mechanisms converge on inhibition of the main adipogenic transcription factors CEBPα and PPARγ. This can be achieved by stabilization of β-catenin which translocates into the nucleus and, binding TCF/LEF transcription factors, facilitates the induction of target genes, such as CCND1, which in turn directly inhibits CEBPα and PPARγ (Christodoulides et al., 2009[13]). Such WNT activation could be caused by WNT1 and WNT3A that were found up-regulated in CF-treated P20 cells (Figure 4(Fig. 4)). In fact, in differentiated 3T3-L1 adipose cells, WNT1 led to inhibition of PPARγ (Grünberg et al., 2014[22]), activating WNT pathways similarly to WNT3A (Gustafson and Smith, 2010[23]). However, in CF-treated P20 cells WNT5B and SFRP5 were also up-regulated (Figure 4(Fig. 4)). WNT5B is known to promote adipogenic process (Nishizuka et al., 2008[42]), while SFRP5 is recognized as WNT protein inhibitor (Bovolenta et al., 2008[6]) which prevents the activation of frizzled receptors and attenuates the noncanonical WNT signaling (Catalán et al., 2014[9]). Its role is still controversial and Wang et al. (2014[61]) considered SFRP5 only a marker of mature adipocytes. Interestingly, treatment of P20 cells with *Rosmarinus officinalis *(Stefanon et al., 2015[54]), *Rhodiola rosea* (Pomari et al., 2015[45]) and hydroxytyrosol (personal communication) activated the WNT signaling and led to the up-regulation of SFRP5 and WNT3A. WNT1 was also up-regulated with hydroxytyrosol treatment, while WNT5B and WNT10B were up-regulated with *Rhodiola rosea* treatment.

The screening of bioactive compounds on pre-adipocytes allowed to rank them for their anti-adipogenic effects. The results in differentiating cells showed that TO was the most active in inducing apoptosis; DHA, CF and RE were more efficient in lowering triglyceride accumulation; CF, DHA and OL sufficiently increased lipolysis. According to these results, the differential expression of an array of genes involved in adipogenesis was analyzed after 20 days of pre-adipocytes treatment with CF and the results indicated a significant up-regulation of genes involved in WNT and GATA signaling and DLK1, which play a relevant role in the inhibition of adipogenesis.

These data suggest that bioactive compounds with anti-adipogenic effects can be considered as potential candidates for further evaluations of their use as nutraceuticals in dietary supplements.

## Acknowledgements

This work was supported by Progetto ART. 13 D.LGS 297/99, Italy.

## Conflict of interest

The authors declare that there are no conflicts of interest.

## Figures and Tables

**Table 1:**
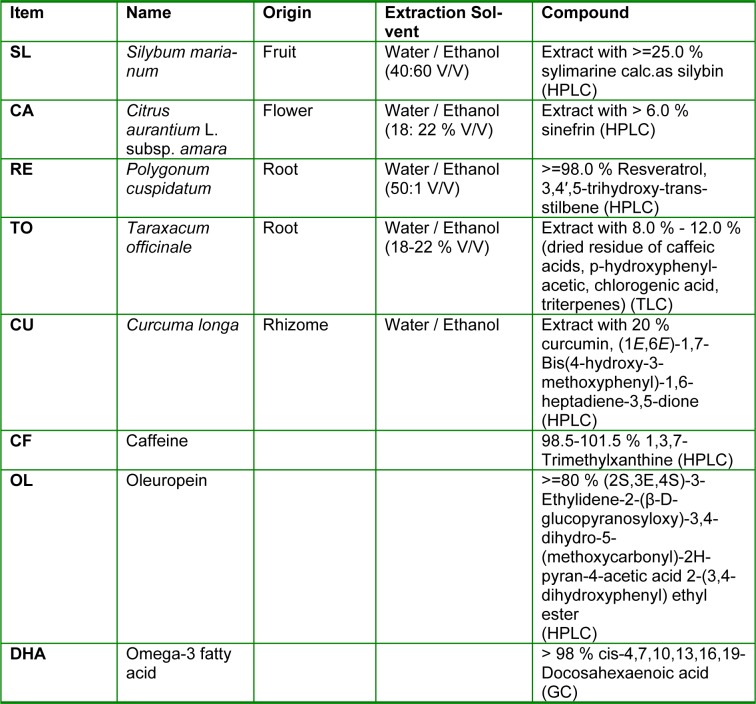
List of compounds used in the study. SL, *Silybum marianum*, CA, *Citrus aurantium*, RE, Resveratrol, TO, *Taraxacum officinale*, CU, *Curcuma longa*, CF, Caffeine, OL, Oleuropein DHA, Docosahexaenoicacid

**Table 2:**
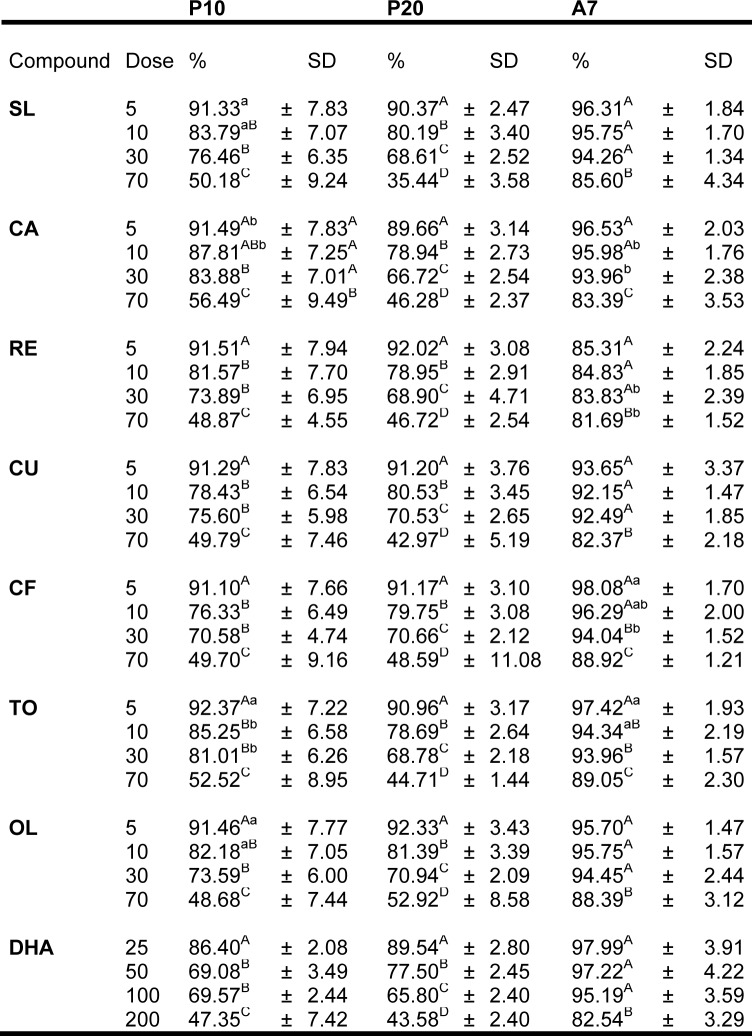
Modulation of MTT metabolism by compounds in human omental pre-adipocytes and adipocytes. Cells were treated with different concentrations of compounds. P10 differentiating pre-adipocytes treated for 10 d; P20 differentiating pre-adipocytes treated for 20 d; A7, mature adipocytes treated for 7 d. Data are expressed as percentage of absorbance in comparison to CTRL cells (untreated) and presented as means ± standard deviation (SD). Different superscript capital letters indicate significant differences (*P* < 0.001) within treatments at different concentrations. Different superscript small letters indicate significant differences (P < 0.05) within treatments at different concentrations. Compound: SL, *Silybum marianum*, CA, *Citrus aurantium*, RE, Resveratrol, TO, *Taraxacum officinale*, CU, *Curcuma longa*, CF, Caffeine, OL, Oleuropein, DHA, Docosahexaenoicacid
